# Supplementary material for: Serum metabolomics identifies unique inflammatory signatures to distinguish rheumatoid arthritis responders and non-responders to TNF inhibitor therapy
Source: Metabolomics. 2025 Aug 12;21(5):112. doi: 10.1007/s11306-025-02310-7 (PMC12343672; doi:10.1007/s11306-025-02310-7)
Supplement: Supplementary file 1 — Supplementary Material 1 [file 11306_2025_2310_MOESM1_ESM.docx]

**Supplementary Data**

**Serum metabolomics identifies unique inflammatory signatures to distinguish rheumatoid arthritis responders and non-responders to TNF inhibitor therapy**

Michele Fresneda Alarcon^1^, Yun Xu^2^, Cassio Lima^2^, Susanna Ford^3^, Rudi Grosman^2,4^, Royston Goodacre^2^, Marie M Phelan^2,4^, Helen L Wright^1^

^1^Institute of Life Course and Medical Sciences, University of Liverpool, Liverpool, L7 8TX, UK

^2^Centre for Metabolomics Research, Department of Biochemistry, Cell and Systems Biology, Institute of Systems Molecular and Integrative Biology, University of Liverpool, Liverpool, L69 7BE, UK

^3^School of Life Sciences, University of Liverpool, Liverpool, L69 7BE, UK

^4^High Field NMR Facility, Liverpool Shared Research Facilities University of Liverpool, Liverpool, L69 7TX, UK

**Supplementary Table 1: Patient demographics.** Data are shown as mean (+/- SD) for each group. RA-R, RA TNFi responders; RA-NR, RA TNFi non-responders. DAS28, 28-joint disease activity score.

|  | RA-R | RA-NR | p-value |
| --- | --- | --- | --- |
| *NMR metabolomics* |  |  |  |
| n | 30 | 8 |  |
| Sex (%F) | 80% | 75% |  |
| Age (years) | 53.5 +/-10.6 | 52.6 +/-11.4 | 0.82 |
| DAS28 | 5.42 +/-0.98 | 5.42 +/-1.12 | 0.78 |
|  |  |  |  |
| *FTIR metabolomics* |  |  |  |
| n | 10 | 10 |  |
| Sex (%F) | 80% | 80% |  |
| Age (years) | 53.5 +/-15.4 | 48.8 +/-12.3 | 0.46 |
| DAS28 | 5.41 +/-0.8 | 5.48 +/-0.8 | 0.84 |

**Supplementary Table 2: Discriminatory Infrared spectral bands, with biomolecular assignments and their bond vibrations**, adapted from (Ghimire et al. 2020; Ramalingam et al. 2014). vas = asymmetric stretching vibration, vs = symmetric stretching vibration, v = v stretching vibration

| Band (cm^-1^) | Assignment and vibrations |
| --- | --- |
| 900-1158 | Carbohydrates (Glucose, Mannose, Fructose) and nucleic acids  (Deoxyribose/Ribose DNA, RNA): C-O, C-C stretch, C-H bends,  Endocyclic C-O-C vibration and, νs(PO^2−^) |
| 1208-1244 | Amide III, νas(PO^2−^) |
| 1317-1382 | Collagen: CH_2_ wagging, the vibration of α, and β anomer |
| 1420-1430 | Polysaccharides, νs (COO-), (CH_2_) |
| 1480-1580 | Amide II of proteins: (α-helical, β-pleated sheet, unordered conformation  structures), δ(N-H), ν(C-N) |
| 1600-1700 | Amide I of proteins: (α-helical, β-pleated sheet, β-turns, random coils, and sidechain, β (anti-‖+turn) structures), ν(C=O), ν(C-N), CNN |
| 1720-1750 | Lipids C=O stretching |

**Supplementary References**

Ghimire, H., X. Hu, G. Qin, A. G. Unil Perera (2020). Optimizing infrared spectral discrimination to enhance disease diagnostics: monitoring the signatures of inflammatory bowel diseases with anti-TNFalpha therapy. Biomed Opt Express 11, 4679-4694 doi:10.1364/BOE.394895

Ramalingam, P., Y. P. Reddy, K. V. Kumar, B. R. Chandu, K. Rajendran (2014). Evaluation of metformin hydrochloride in Wistar rats by FTIR-ATR spectroscopy: A convenient tool in the clinical study of diabetes. J Nat Sci Biol Med 5, 288-92 doi:10.4103/0976-9668.136168
